# Characteristics of CYP3A4-related potential drug-drug interactions in outpatients receiving prescriptions from multiple clinical departments

**DOI:** 10.1186/s40780-024-00368-4

**Published:** 2024-08-05

**Authors:** Rina Matsuoka, Shinsuke Akagi, Tomohiro Konishi, Masashi Kondo, Hideki Matsubara, Shohei Yamamoto, Keiji Izushi, Yuichi Tasaka

**Affiliations:** 1 Laboratory of Clinical Pharmacy, School of Pharmacy, Shujitsu University, 1-6-1 Nishigawara, Naka-Ku, Okayama, 703-8516 Japan; 2 Kojima Ai Pharmacy, 2-19 Kojimaekimae, Kurashiki, Okayama 711-0921 Japan; 3 Uizu Pharmacy, 1-1-9 Kojimaajino, Kurashiki, Okayama 711-0913 Japan; 4 Fuji Pharmacy, 2-7-25 Kojimaajinokami, Kurashiki, Okayama 711-0917 Japan; 5 Koukando Pharmacy, 1-1-15 Kojimaajino, Kurashiki, Okayama 711-0913 Japan; 6 Izushi Pharmacy, 1-88 Kojimaekimae, Kurashiki, Okayama 711-0921 Japan

**Keywords:** Drug-drug interaction, CYP3A4, Multiple clinical department prescriptions, Adverse drug reactions

## Abstract

**Background:**

Drug-drug interactions (DDIs) increase the incidence of adverse drug reactions (ADRs). In a previous report, we revealed that the incidence of potential DDIs due to the same CYP molecular species in one prescription exceeds 90% among patients taking six or more drugs and that CYP3A4 markedly influences the increase in the number of potential DDIs in clinical practice. However, the factors contributing to an increased number of potential DDIs in prescriptions from multiple clinical departments remain poorly clarified.

**Methods:**

This observational study was performed at five pharmacies in Okayama Prefecture, Japan. Patients who visited these pharmacies from 11 April 2022 to 24 April 2022 were included, except those who had prescriptions only from a single clinical department. A stratified analysis was performed to determine the incidence of CYP3A4-related potential DDIs according to the number of drugs taken. Additionally, factors associated with an increase in the number of drugs involved in CYP3A4-related potential DDIs were identified using multiple linear regression analysis. In this study, potential DDIs for the prescription data subdivided by clinical department, containing two or more drugs, were used as control data.

**Results:**

Overall, 372 outpatients who received prescriptions from multiple clinical departments were included in the current study. The number of drugs contributing to CYP3A4-related potential DDIs increased with an increase in the number of clinical departments. Notably, in cases taking fewer than six drugs, prescriptions from multiple clinical departments had a higher frequency of CYP3A4-related potential DDIs than those in prescriptions subdivided by clinical department. Multiple regression analysis identified "Cardiovascular agents", "Agents affecting central nervous system", and "Urogenital and anal organ agents" as the top three drug classes that increase CYP3A4-related potential DDIs.

**Conclusion:**

Collectively, these results highlight the importance of a unified management strategy for prescribed drugs and continuous monitoring of ADRs in outpatients receiving prescriptions from multiple clinical departments even if the number of drugs taken is less than six.

## Background

The effective management of adverse drug reactions (ADRs) is vital for improving the quality of pharmacotherapy. A meta-analysis has shown that drug-related deaths in hospitalized patients account for 5.6% of all deaths, half of which are preventable [1]. Additionally, ADRs account for approximately 5% of hospital admissions [2–5]. Drug-drug interactions (DDIs) are also a notable cause of hospital admissions and visits [6]. A study analyzing a spontaneous reporting database has revealed that approximately one-third of patients exposed to a potential DDI actually experienced a serious ADR [7]. Moreover, a retrospective cohort study has demonstrated that most ADRs resulting in death among hospitalized patients originally occurred outside of the hospital and that DDIs occurred in 44% of drug-related deaths [8]. Accordingly, evidence regarding factors associated with an increased number of DDIs in outpatients would be beneficial in improving the quality of ADR monitoring for the early detection of ADR signals and avoidance of severe ADRs.

DDIs can be classified into pharmacokinetic interactions, which cause changes in the blood concentrations of drugs, and pharmacodynamic interactions, which do not involve changes in blood concentrations [9]. Despite the diverse mechanisms underlying DDIs, approximately 40% of DDIs are pharmacokinetic interactions, most of which are thought to involve CYP molecular species [10]. In addition, DDIs become more complex as more drugs are associated with the same CYP molecular species. Moreover, although the co-administration of certain drugs is classified in contraindicated or precautionary based on the risk–benefit balance, the safety of DDIs due to three or more drugs interacting with the same CYP molecular species remains poorly explored. However, a retrospective study covering 10 years revealed that approximately 50% of DDI-related ADRs were preventable [11]. Therefore, switching to a combination with fewer potential DDIs or monitoring potential ADRs due to DDIs is one of the clinical practices through which pharmacists can exercise their professional competence for improving drug safety.

We previously reported that the incidence of potential DDIs due to the same CYP molecular species exceeds 90% among patients taking six or more drugs and that CYP3A4 has a substantial impact on the increased number of potential DDIs in outpatients [12]. However, because this previous report was limited to a single prescription, knowledge of potential DDIs in prescriptions from multiple clinical departments in the community, including factors responsible for the increased number of potential DDIs, is poorly elucidated. Accumulating evidence on these current conditions could contribute to improving the quality and safety of medications associated with DDIs in outpatients. Thus, we examined the incidence of potential DDIs and factors associated with an increase in the number of drugs involved in potential DDIs in prescriptions from multiple clinical departments, focusing on CYP3A4, which markedly contributes to potential DDIs.

## Methods

### Data collection

This observational study was performed at five pharmacies (Kojima Ai Pharmacy, Uizu Pharmacy Kojima, Fuji Pharmacy, Koukando Pharmacy Ajino, and Izushi Pharmacy) in Okayama Prefecture, Japan. Patients who visited these pharmacies from April 11, 2022, to April 24, 2022, were included, except those prescribed therapy by a single clinical department. The survey items included age, sex, name of the medical institution, clinical departments issuing the prescriptions, and prescribed drugs. These data were obtained from the prescription itself, drug history accumulated in the pharmacy, or medication notebook and transcribed using Microsoft Excel in each pharmacy. Data analysis was performed at Shujitsu University. Topical medications were excluded from data collection.

### Definition of potential DDIs

The metabolic pathway of each drug was investigated using the package insert and an interview form for each brand-name drug. Combination drugs were analyzed separately for each active ingredient (e.g., a combination drug containing two active ingredients was analyzed as two drugs). However, for combination drugs in which multiple active ingredients were not marketed separately, the analysis was conducted as one drug for each formulation (e.g., tegafur, gimeracil, and oteracil potassium). The involvement of CYP3A4 in the metabolic process was classified as “inhibition,” “competition” (including the description of “substrate”), or “induction (enzyme induction).” These categories include drugs for which 3A4 is not a major CYP involved in the metabolism of the drug in question, and the degree of influence may not be significant. It is difficult to conclude that these drugs do not affect metabolism by CYP3A4 based solely on the information provided in the package insert and interview form. Thus, in the current study, “potential DDI” in the metabolic process was defined as the presence of an “inhibition” drug and a “competition” drug or more than two “competition” drugs among prescribed drugs. Given that the effect of the “induction” drug on “competition” and “inhibition” drugs was unclear from the information in the package insert or interview form alone, the involvement of the “induction” drug was excluded from the analysis. These methods follow those used in a previous study [12].

### Statistical analysis

Herein, prescriptions from the same clinical department of another medical institution were analyzed as prescriptions from other clinical departments. In addition, potential DDIs for the subdivided prescription data by clinical department, which containing two or more of drugs, were used as control data. For analyzing differences between the two groups, the Mann–Whitney *U* and chi-square tests were used for continuous variables and frequencies, respectively. Threshold values for the number of drugs taken were calculated using a receiver operating characteristic (ROC) curve (STATA 18 [StataCorp, LLC., TX, USA]). In addition, we calculated the sensitivity and specificity of the threshold value and the area under the ROC curve (AUC). Multiple linear regression analysis was performed to identify factors associated with an increase in the number of drugs involved in CYP3A4-related potential DDIs. The objective variable was the number of drugs involved in CYP3A4-related potential DDIs, and the explanatory variables were age, sex (0: male, 1: female), number of clinical departments, and the number of drugs taken per therapeutic category. Multicollinearity in the multiple regression analysis was assessed using the variance inflation factor (VIF). In the preliminary analysis, although the number of drugs taken was strongly associated with potential DDIs, the VIF values suggested the possibility of confounding other explanatory variables. Thus, multiple regression analysis was conducted without the number of drugs taken as an explanatory variable. Statistical analyses for the Mann–Whitney U test, chi-square test, and multiple regression analysis were performed using IBM SPSS Statistics 25 (Japan IBM, Ltd., Tokyo, Japan), and statistical significance was set at P < 0.05. Therapeutic category was classified according to the therapeutic category of drugs in Japan [13].

### Ethical approval

This study was approved by the Ethical Review Committee of Shujitsu University Shujitsu Junior College (approval no. 255) and conducted in compliance with the Ethical Guidelines for Medical and Biological Research Involving Human Subjects.

## Results

### Characteristics of the patients

A total of 372 patients were included in the current study, with 142 males (38.2%) and 230 females (61.8%). Table 1 summarizes the patient characteristics. The most common age groups were 80–89 (37.4%), 70–79 (34.4%), and 60–69 (8.6%), and patients aged ≥ 65 accounted for 85.8% of all patients. The average number of drugs taken was 7.2. Overall, 81.2%, 17.5%, and 1.3% of patients had visited two, three, and four clinical departments, respectively.

**Table 1:** Characteristics of the patients

	Frequency and percentage of cases	
a) Sex		
Male	142	38.2%
Female	230	61.8%
Overall	372	100.0%
b) Age (years)		
0–9	0	0.0%
10–19	1	0.3%
20–29	3	0.8%
30–39	6	1.6%
40–49	8	2.2%
50–59	24	6.5%
60–69	32	8.6%
70–79	128	34.4%
80–89	139	37.4%
90 ≤	31	8.3%
Overall	372	100.0%
65 ≤	319	85.8%
c) Number of drugs taken		
2–3	23	6.2%
4–5	105	28.2%
6–7	93	25.0%
8–9	76	20.4%
10 ≤	75	20.2%
Overall	372	100.0%

### Incidence of CYP3A4-related potential DDIs and number of drugs involved

**Fig. 1:**
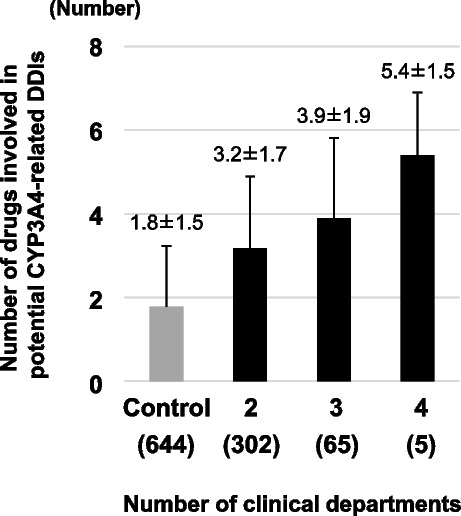
Number of drugs involved in CYP3A4-related potential DDIs. Prescriptions subdivided by clinical department, containing two or more drugs, were used as control data for the number of drugs involved in CYP3A4-related potential DDIs (involving two or more drugs). Numbers of patients are indicated n parentheses. DDIs, drug-drug interactions. Values are shown as means ± standard deviation

Of the 372 eligible patients, 322 (86.6%) had potential DDIs involving CYP3A4. The incidence of potential DDIs increased with the number of clinical departments visited. The incidence of potential DDIs of subdivided control data was 52.5%. However, the incidence of potential DDIs increased to 84.8%, 93.8%, and 100.0% with two, three, and four clinical departments, respectively. The number of drugs involved in potential DDIs tended to increase with the number of clinical departments: 1.8 ± 1.5 drugs in the subdivided control data, and 3.2 ± 1.7, 3.9 ± 1.9, and 5.4 ± 1.5 drugs with two, three, and four clinical departments, respectively (Fig. 1). The number of drugs taken and the CYP3A4-related potential DDIs were significantly higher in the multiple clinical department group than that in the subdivided control data, both in the group taking ˃6 drugs and in the group taking < 6 drugs. On the other hand, the incidence of CYP3A4-related potential DDIs due to ≥ 2 or ≥ 3 drugs was significantly higher in the multiple clinical department group than those in the subdivided control data only when the number of drugs taken was < 6 (Table 2). An ROC curve analysis investigating the existence of potential CYP3A4-related DDIs in prescriptions from multiple clinical departments, which was associated with the number of drugs taken, was also performed. The calculated threshold value of the number of drugs taken was six (sensitivity: 0.58, specificity: 0.90, AUC: 0.82 [95% confidence interval (CI): 0.77–0.88]) and six (sensitivity: 0.69, specificity: 0.82, AUC: 0.82 [95% CI: 0.78–0.87]) for potential CYP3A4-related DDIs due to ≥ 2 or ≥ 3 drugs, respectively.

**Table 2:** Incidence of CYP3A4-related potential DDIs by number of drugs taken

	Number of drugs taken	Subdivided control data^†^	Multiple clinical departments	*P*-value
Number of patients	less than 6	516	128	
	6 or more	128	244	
Number of drugs taken	less than 6	3.0 ± 1.0	4.3 ± 0.8	< 0.01*^, a^
	6 or more	7.5 ± 1.7	8.7 ± 2.4	< 0.01*^, a^
Number of drugs involved CYP3A4-related potential DDIs	less than 6	1.4 ± 1.0	2.1 ± 1.2	< 0.01*^, a^
	6 or more	3.5 ± 1.6	4.0 ± 1.7	< 0.01*^, a^
Incidence of CYP3A4-related potential DDI due to two or more drugs	less than 6	43.2%	71.1%	< 0.01*^, b^
	6 or more	89.8%	94.7%	0.090^b^
Incidence of CYP3A4-related potential DDI due to three or more drugs	less than 6	13.6%	35.2%	< 0.01*^, b^
	6 or more	74.2%	81.2%	0.142^b^

Values for the number of drugs is shown as the mean ± standard deviation

*DDIs* Drug-drug interactions

^†^Prescriptions subdivided by clinical department, which containing two or more of drugs, were used as control data for the number of drugs involved and incidence of CYP3A4-related potential DDIs

^*^P < 0.05, ^a^Mann-Whitney *U* test, ^b^chi-square test

### Factors associated with an increase in the number of drugs involved in CYP3A4-related potential DDIs

Multiple linear regression analysis was performed to identify factors responsible for an increase in the number of CYP3A4-related potential DDIs in prescriptions from multiple clinical departments. Agents affecting central nervous system, cardiovascular agents, digestive organ agents, urogenital and anal organ agents, blood and body fluid agents, other agents affecting metabolism, allergic agents, and alkaloidal narcotics were extracted as factors that increased the number of drugs involved in CYP3A4-related potential DDIs (Table 3). Additionally, the analysis by *t*-value showed that the top three therapeutic categories with the highest impact on CYP3A4-related potential DDIs were cardiovascular agents (*t* = 10.847), agents affecting central nervous system (*t* = 9.038), and urogenital and anal organ agents (*t* = 7.603).

**Table 3:** Factors associated with an increase in the number of drugs involved in CYP3A4-related potential DDIs

	B	SE	b	*t*-value	*p*-value	VIF
Age	-0.004	0.005	-0.030	-0.737	0.461	1.316
Sex (0: male, 1: female)	-0.270	0.142	-0.076	-1.905	0.058	1.255
Number of clinical departments	0.235	0.157	0.059	1.499	0.135	1.215
Agents affecting central nervous system	0.425	0.047	0.352	9.038	< 0.01*	1.200
Agents affecting peripheral nervous system	-0.443	0.332	-0.049	-1.335	0.183	1.063
Agents affecting sensory organs	-0.052	0.262	-0.007	-0.200	0.842	1.037
Cardiovascular agents	0.515	0.048	0.450	10.847	< 0.01*	1.362
Respiratory organ agents	0.104	0.139	0.029	0.753	0.452	1.209
Digestive organ agents	0.247	0.058	0.168	4.250	< 0.01*	1.230
Hormones	0.280	0.172	0.061	1.631	0.104	1.121
Urogenital and anal organ agents	0.861	0.113	0.330	7.603	< 0.01*	1.493
Vitamins	-0.123	0.097	-0.049	-1.275	0.203	1.179
Nutrients, tonics	0.063	0.298	0.008	0.212	0.832	1.094
Blood and body fluid agents	0.623	0.161	0.149	3.868	< 0.01*	1.169
Other agents affecting metabolism	0.309	0.058	0.209	5.289	< 0.01*	1.231
Antineoplastics	0.366	0.295	0.045	1.243	0.215	1.054
Allergic agents	0.543	0.113	0.198	4.808	< 0.01*	1.348
Traditional Chinese medicines	-0.284	0.257	-0.040	-1.102	0.271	1.054
Antibiotics	0.585	0.250	0.095	2.345	0.020*	1.292
Chemotherapeutics	0.533	0.508	0.039	1.051	0.294	1.079
Alkaloidal narcotics	1.180	0.548	0.078	2.152	0.032*	1.052

*DDIs* Drug-drug interactions, *B* Partial regression coefficient, *SE* Standard error, *b* Standardized partial regression coefficient, *VIF* Variance inflation factor

^*^P < 0.05

Among prescribed drugs, 23 of 67 (34.3%) cardiovascular drugs were CYP3A4-related agents, contributing to CYP3A4-related DDIs in 360 out of a total of 680 prescriptions (52.9%). The top 5 drugs by the number of prescriptions were as follows: amlodipine besilate, 127; rosuvastatin calcium, 75; nifedipine, 30; bisoprolol fumarate, 29; and atorvastatin calcium hydrate, 25. Likewise, 38 of 80 (47.5%) agents affecting central nervous system were CYP3A4-related drugs, contributing to potential DDIs in 259 out of a total of 519 prescriptions (49.9%). The top 5 drugs by number of prescriptions were as follows: celecoxib, 42; brotizolam, 28; acetaminophen, 26; etizolam, 29; and trazodone hydrochloride, 13. Notably, 8 of 10 (80.0%) urogenital and anal organ agents were CYP3A4-related drugs, contributing to potential DDIs in 126 out of a total of 135 prescriptions (93.3%). The top 5 drugs by number of prescriptions were as follows: mirabegron, 46; naftopidil, 23; tamsulosin hydrochloride, 18; vibegron, 16; and sirodosin, 9 (Table 4). Additionally, 9 out of a total of 680 cardiovascular agent prescriptions (1.3%) and 9 out of a total of 519 prescriptions of agents affecting central nervous system (1.7%) were prescribed by urology, whereas 90.4% (122 out of a total of 135) urogenital and anal organ agents were prescribed by urologists.

**Table 4:** Urogenital and anal organ agents prescribed to the patients

Objective drug	Number of cases	CYP3A4 competitive or inhibitory action
Mirabegron	46	YES
Naftopidil	23	YES
Tamsulosin hydrochloride	18	YES
Vibegron	16	YES
Silodosin	9	YES
Imidafenacin	8	YES
Fesoterodine fumarate	7	YES
Melilot extract	4	NO
Solifenacin succinate	3	YES
Cernitin pollen extract	1	NO

## Discussion

Although the early detection of DDI-induced ADRs can contribute to affording safe drug therapy, there is limited information on drugs capable of increasing DDIs in clinical practice for utilization by community pharmacists. Therefore, in the current study, we retrospectively investigated the occurrence of potential DDIs in patients who received prescriptions from multiple clinical departments.

The average number of drugs taken was 7.2, which was 1.6-fold higher than the 4.5 drugs per prescription in the same region reported in our previous study [12]. Additionally, in the current study, the incidence of CYP3A4-related potential DDIs with prescriptions from multiple clinical departments (84.8–100.0%) was 1.6–1.9-fold higher than that of the subdivided control data (52.5%). These results indicate the importance of centralized drug management from the perspective of DDIs for patients who receive prescriptions from multiple clinical departments. Although 81.2% of patients visited two clinical departments in the current study, an analysis based on receipt data from more than 1 million patients in Tokyo has revealed that the sum of patients who visit three or four medical institutions does not differ from the proportion who visit two medical institutions among those aged ≥ 75 years [14]. This discrepancy may be attributed to differences in the methodology of this study, which was counted by the clinical department, not by a medical institution, or conducted in one local city area, the Kojima region, Okayama. However, the receipt-based study discussed above supports the significance of focusing on prescriptions from multiple clinical departments.

Previous study has reported a substantially higher incidence of ADRs in hospitalized patients taking ≥ 6 drugs when compared with those taking 1–3 drugs [15]. Thus, in the current study, eligible patients were divided into two groups for analysis: those taking ≥ 6 drugs and those taking ≤ 6 drugs. The incidence of CYP3A4-related potential DDIs due to two or more or three or more drugs was markedly elevated only in patients taking less than six drugs in the multiple clinical department group when compared with the subdivided control data. This finding may suggest that DDI-related ADRs require more attention in patients receiving prescriptions from multiple clinical departments than in those receiving prescriptions from a single department, even if the number of drugs taken is less than six. Additionally, given that metabolic mechanisms via CYP molecular species become more complex, DDIs due to three or more drugs may exert a greater impact on drug blood levels and the occurrence of ADRs than DDIs due to two drugs. Herein, the 2.6-fold higher incidence of CYP3A4-related potential DDIs due to three or more drugs in patients taking less than six drugs in multiple clinical department group, compared with that in the subdivided control data, emphasizes the importance of centralized management of multiple clinical department prescriptions. In contrast, when six or more drugs were taken, there was no difference in the frequency of potential DDIs between the subdivided control data and the prescription data from multiple clinical departments. Polypharmacy is a well-recognized risk factor for DDIs [16, 17]. Our ROC curve analysis also showed that the calculated threshold values of the number of drugs taken, which was associated with the existence of potential CYP3A4-related DDIs, were both six drugs in potential CYP3A4-related DDIs due to ≥ 2 or ≥ 3 drugs in the prescriptions from multiple clinical departments. Although these results support our decision to divide the data into more than and less than six drugs, the findings of this study highlight that attention should be paid to ADRs caused by CYP3A4-related potential DDIs in patients taking over six drugs, regardless of the number of prescribing clinical departments. One study has reported that the use of potentially inappropriate medications is not necessarily associated with the number of drugs prescribed [18]. Therefore, in patients with polypharmacy, in addition to reducing the number of drugs, it is crucial to focus on optimizing the content of prescriptions from the perspective of safety, including DDIs.

Multiple regression analysis identified the top three therapeutic categories with the highest impact on CYP3A4-related potential DDIs, i.e., cardiovascular agents, agents affecting central nervous system, and urogenital and anal organ agents. Although the number of prescriptions for urogenital and anal organ agents was lower than that for cardiovascular agents or agents affecting central nervous system, most of prescribed urogenital and anal organ agents were CYP3A4-related drugs. Considered in conjunction with the finding that more than 90% of urogenital and anal organ agents are prescribed in urology, whereas most cardiovascular agents and agents affecting central nervous systems are not prescribed in urology, urogenital and anal organ agents may increase the risk of ADRs due to potential CYP3A4-related DDIs that prescribing doctors are not always aware of in patients receiving prescriptions from multiple clinical departments. These findings would be useful in alerting community pharmacists to the existence of CYP3A4-related potential DDIs, and also highlight the importance of centralized management of prescription drugs by community pharmacists. Patients are more likely to experience potential DDIs and serious DDIs with increasing age [19, 20]. However, as 85.8% of the patients in this study were aged ≥ 65 years or older, “age” may have been overlooked as a factor contributing to the increased number of potential DDIs. In addition, the number of clinical departments was not extracted as a factor influencing the increase in potential DDIs by multiple regression analysis in this study. This may be because the number of patients who received prescriptions from three or four clinical departments was smaller than the number of patients who received prescriptions from two departments. A survey in community pharmacies in Croatia revealed that 84% of eligible patients experienced at least one clinically notable DDI [21]. A cross-sectional and cohort study in Denmark has reported that CYP3A4 is the most prominent CYP isoenzyme involved in mortality and readmission rates; the authors also found that combinations of clarithromycin with ticagrelor, tacrolimus, or everolimus were associated with substantially elevated readmission rates [22]. Thus, the optimization of prescriptions to reduce the potential DDIs is of great importance in reducing the risk of ADRs occurrence. Future research on the occurrence of ADRs due to CYP3A4-related DDI with a focus on combinations with high-risk drugs (e.g., antiplatelet agents and immunosuppressant drugs) may be needed, in addition to exploring the number of drugs involved in CYP3A4-related potential DDIs.

This study has several limitations. First, because we identified patients who were prescribed drugs from multiple clinical departments based on their prescription itself, drug history accumulated in the pharmacy, or medication notebook, all patients who were prescribed drugs from multiple clinical departments may not have been identified. Second, because this study was conducted in five pharmacies within the same region, the drugs included in the analysis may reflect the characteristics of the region. Third, we excluded the intensity affecting metabolism by CYP3A4. Although concomitant use of drugs metabolized by the same CYP molecular species (potential DDIs by more than only two “competition” drugs in this study) is often listed as a concomitant use precaution in the package insert, the actual impact on DDI has not been fully investigated. We also excluded the involvement of CYP3A4-inducing drugs. Of the 372 patients, 7 (1.9%) were prescribed some CYP3A4-inducing drugs, and actual DDIs may differ from our results. The same limitation would be applicable to metabolic pathways other than CYP3A4. Finally, we did not examine the presence of ADRs. Further clinical studies are required to determine the extent to which CYP3A4-related potential DDIs result in ADRs. Nevertheless, the results of the current study highlight the importance of centralized drug management for patients who receive prescriptions from multiple clinical departments to avoid DDIs. Furthermore, ADR monitoring utilizing the results of this study would contribute to the early detection of DDI-induced ADRs in an aging society.

## Conclusions

Prescriptions from multiple clinical departments exhibit a markedly higher incidence of CYP3A4-related potential DDIs than those subdivided by clinical department when the number of drugs taken is less than six. Additionally, cardiovascular agents, agents affecting central nervous system, and urogenital and anal organ agents were identified as the top three therapeutic categories involved in the increase in CYP3A4-related potential DDIs. These findings suggest the need for centralized management of prescription drugs in outpatients and provide useful insights for community pharmacists in the management of ADR caused by CYP3A4-related DDIs.

## Data Availability

Not applicable.

## Abbreviations

ADRs: Adverse drug reactions
DDIs: Drug-drug interactions
